# Biochemical Effects of *Petroselinum crispum* (Umbellifereae) Essential Oil on the Pyrethroid Resistant Strains of *Aedes aegypti* (Diptera: Culicidae)

**DOI:** 10.3390/insects10010001

**Published:** 2018-12-24

**Authors:** Jitrawadee Intirach, Anuluck Junkum, Nongkran Lumjuan, Udom Chaithong, Pradya Somboon, Atchariya Jitpakdi, Doungrat Riyong, Danita Champakaew, Roongtawan Muangmoon, Arpaporn Chansang, Benjawan Pitasawat

**Affiliations:** 1 Center of Insect Vector Study, Department of Parasitology, Faculty of Medicine, Chiang Mai University, Chiang Mai 50200, Thailand; tangmo.parasite@gmail.com (J.I.); udom.c@cmu.ac.th (U.C.); pradya.somboon@cmu.ac.th (P.S.); ajitpakdi2@yahoo.com (A.J); doungrat.riyong@cmu.ac.th (D.R.); ruda_0@hotmail.com (D.C.); belle_roong@hotmail.com (R.M.); ar.chansang@gmail.com (A.C.); benjawan.p@cmu.ac.th (B.P.); 2 Graduate PhD’s Degree Program in Parasitology, Faculty of Medicine, Chiang Mai University, Chiang Mai 50200, Thailand; 3 Research Institute for Health Sciences, Chiang Mai University, Chiang Mai 50200, Thailand; nklumjuan@yahoo.com

**Keywords:** *Aedes aegypti*, enzyme activity, esterases, mixed-function oxidases, *Petroselinum crispum*, phosphatases

## Abstract

In ongoing screening research for edible plants, *Petroselinum crispum* essential oil was considered as a potential bioinsecticide with proven antimosquito activity against both the pyrethroid susceptible and resistant strains of *Aedes aegypti*. Due to the comparative mosquitocidal efficacy on these mosquitoes, this plant essential oil is promoted as an attractive candidate for further study in monitoring resistance of mosquito vectors. Therefore, the aim of this study was to evaluate the impact of *P. crispum* essential oil on the biochemical characteristics of the target mosquito larvae of *Ae. aegypti*, by determining quantitative changes of key enzymes responsible for xenobiotic detoxification, including glutathione-*S*-transferases (GSTs), *α*- and *β*-esterases (*α*-/*β*-ESTs), acetylcholinesterase (AChE), acid and alkaline phosphatases (ACP and ALP) and mixed-function oxidases (MFO). Three populations of *Ae. aegypti*, comprising the pyrethroid susceptible Muang Chiang Mai-susceptible (MCM-S) strain and the pyrethroid resistant Pang Mai Dang-resistant (PMD-R) and Upakut-resistant (UPK-R) strains, were used as test organisms. Biochemical study of *Ae. aegypti* larvae prior to treatment with *P. crispum* essential oil revealed that apart from AChE, the baseline activity of most defensive enzymes, such as GSTs, *α*-/*β*-ESTs, ACP, ALP and MFO, in resistant UPK-R or PMD-R, was higher than that determined in susceptible MCM-S. However, after 24-h exposure to *P. crispum* essential oil, the pyrethroid susceptible and resistant *Ae. aegypti* showed similarity in biochemical features, with alterations of enzyme activity in the treated larvae, as compared to the controls. An increase in the activity levels of GSTs, *α*-/*β*-ESTs, ACP and ALP was recorded in all strains of *P. crispum* oil-treated *Ae. aegypti* larvae, whereas MFO and AChE activity in these mosquitoes was decreased. The recognizable larvicidal capability on pyrethroid resistant *Ae*. *aegypti*, and the inhibitory effect on AChE and MFO, emphasized the potential of *P. crispum* essential oil as an attractive alternative application for management of mosquito resistance in current and future control programs.

## 1. Introduction

The mosquito, *Aedes* (*Stegomyia*) *aegypti* (Linnaeus, 1762), is an important vector for transmitting life-threatening human diseases like dengue, chikungunya, Zika, and yellow fever in tropical and subtropical regions worldwide [1,2,3]. Dengue is the World’s most critical mosquito-borne viral disease, which inflicts millions of deaths each year. Approximately 3.9 billion people, in 128 countries, are currently at risk of dengue infection, with an estimated 50–100 million and 250,000–500,000 cases of dengue fever and dengue hemorrhagic fever, respectively [4,5]. The number of dengue cases reported annually to the World Health Organization (WHO) has increased from 0.4 to 1.3 million in the decade 1996 to 2005, reaching 2.2, 3.2, and 3.8 million in 2010, 2015, and 2016, respectively [2,6,7]. Since several dengue vaccine candidates are currently in the developmental stage, there is no specific treatment or vaccine commercially available. Therefore, personal protection, environmental management, and mosquito control remain the most important tools in preventing and controlling transmission of this disease [5,8,9,10].

Management of mosquito vectors by applying appropriate chemical compounds such as larvicides, adulticides, attractants, deterrents, and repellents is an essential element to minimize disease transmission [11,12,13]. At least four main classes of synthetic insecticides; namely organochlorines (exclusively DDT), organophosphates, carbamates, and pyrethroids have been in use over decades in vector control programs. In addition to low mammalian toxicity, synthetic pyrethroids have become the most popular and prevalent active ingredients in public health vector control programs, due to their high invertebrate potency at low levels, resulting in rapid immobilization (‘knockdown’) and killing of mosquitoes [14,15]. Although synthetic substances that dramatically reduce the risk of vector-borne diseases have been documented, the overuse and misuse of conventional chemicals, for example, pyrethroids and other insecticides, have led to mosquito resistance, which threatens the potentiality of vector control [16,17,18,19]. A recommended approach for monitoring and managing insecticide resistance in mosquito populations is to search for alternative interventions in order to restrain or reduce the evolution of further resistance as well as preserve the efficiency of existing insecticides [19,20]. Use of biopesticides is encouraged as a promising economic and eco-friendly strategy for fighting the resistance problem [21,22]. Plant essential oils with insecticidal activity have been developed as pesticides for many years, and some have been used commercially as pesticides or repellents against garden and household pests. However, their use as mosquito larvicides is limited, due to their relatively low toxicity against mosquito larvae [22,23,24]. In consequence, research and development of new natural alternatives have been designed and performed by focusing on the improvement of their biological activity [25,26,27]. A literature survey revealed that there are several studies elucidating the behavioral responses or biochemical constituents of target mosquitoes, which could enable a better understanding of plant essential oil effects and their possible mode of action [28,29,30]. 

As part of the ongoing screening research for local edible plants in Thailand, *Petroselinum crispum* fruit oil was considered as a potential bioinsecticide, with proven antimosquito activity against both pyrethroid susceptible and resistant strains of *Ae. aegypti* [31]. The larvicidal potential of *P. crispum* oil is effective, with an insignificant difference of LC_50_ values of 43.22, 44.50 and 44.03 ppm against Muang Chiang Mai-susceptible (MCM-S), Pang Mai Dang-resistant (PMD-R), and Upakut-resistant (UPK-R) strains of *Ae. aegypti*, respectively. The GC-MS analysis of *P. crispum* oil, which is an identical sample used in this study, revealed the presence of 19 bioactive compounds, accounting for 98.25% of the whole oil, with the principal constituents being thymol (42.41%), *p*-cymene (27.71%) and γ-terpinene (20.98%) [31]. Despite *P. crispum* oil producing less larvicidal potential than synthetic insecticides such as temephos, permethrin, and deltamethrin; the comparative efficacy on pyrethroid susceptible and resistant *Ae. aegypti* makes it an attractive candidate for further research on monitoring resistance in mosquito vectors. As the essential oil is a mixture of bioactive materials that functions through the different modes of actions [32,33], *P. crispum* oil potentially reduces the risk of resistance development in mosquitoes. The literature, however, offers no data about the insect detoxification mechanisms and biochemical responses to this plant essential oil.

Major mechanisms of pyrethroid resistance in insects involve mutation within the target site of the insecticide and/or increase in the rate of insecticide detoxification. Metabolic detoxification of insecticides is associated with alterations in the activity of defensive enzymes such as glutathione-*S*-transferases (GSTs), *α*- and *β*-esterases (*α*-/*β*-ESTs), acetylcholinesterase (AChE), acid and alkaline phosphatases (ACP and ALP), and mixed-function oxidases (MFO), which help organisms to transform and/or eliminate endogenous and exogenous compounds [34,35,36,37]. An understanding of biochemical changes to plant-derived product, such as *P. crispum* essential oil, plays a vital role in vector management, especially resolution of insecticide resistance. Therefore, the aim of this study was to evaluate the biochemical effects of the *P. crispum* essential oil caused in *Ae. aegypti* strains. Quantitative changes of important marker enzymes responsible for xenobiotic detoxification, including GSTs, *α*-/*β*-ESTs, AChE, ACP, ALP, and MFO, were determined in either the susceptible or resistant strain of *Ae. aegypti* larvae treated with *P. crispum* essential oil. The information obtained from this study has enhanced basic knowledge and useful guidelines on how to use and improve plant products for future management of vector control in areas with high levels of pyrethroid resistance.

## 2. Materials and Methods

### 2.1. Preparation of P. crispum Oil

Dried fruit of *P. crispum* was obtained commercially from medicinal plant suppliers in Chiang Mai province, Thailand. Scientific identification of this plant specimen was accomplished by Miss Wannaree Charoensup, a scientist at the Department of Pharmaceutical Science, Faculty of Pharmacy, Chiang Mai University (CMU). A reference voucher, PARA-PE-001-Fr/1, was deposited at the Department of Parasitology, Faculty of Medicine, CMU. The identified *P. crispum* fruit was shade-dried for one week in an open area, with active ventilation and ambient temperature of about 30 ± 5 °C, in order to remove the moisture content before extracting essential oil. After grinding, the fruit powder of *P. crispum* was subjected to extractions by steam distillation at 100 °C for at least 3 h. The essential oil obtained was collected, dried over anhydrous sodium sulfate (Na_2_SO_4_) to make it moisture free, and preserved in an airtight brown bottle at 4 °C in a refrigerator until its use in experimental bioassays.

### 2.2. Chemicals and Reagents

The albumin standard Thermo Fisher Scientific (Waltham, MA, USA) and Bio Rad protein reagent (Bio-Rad Laboratories, Bercules, CA, USA) were used to determine total protein. Cytochrome c from bovine heart (≥95%) (Sigma-Aldrich, St. Louis, MO, USA), *α*-naphthol (≥99%) (Sigma-Aldrich, Buchs, SG, Switzerland), *β*-naphthol (99%) (Sigma-Aldrich, Shanghai, China), and *p*-nitrophenol (99%) (Sigma-Aldrich, St. Louis, MO, USA) were used in the biochemical assays of MFO, *α*-/*β*-ESTs, and ACP/ALP, respectively. The substrates of enzyme analysis, including 3,3′,5,5′-tetramethylbenzidine dihydrochloride hydrate powder (≥98%) (Sigma-Aldrich, Buchs, SG, Switzerland), *α*-naphthyl acetate (≥98%) (Sigma-Aldrich, Buchs, SG, Switzerland), *β*-naphthyl acetate (98%) (Sigma-Aldrich, Buchs, SG, Switzerland), 1-chloro-2, 4-dinitrobenzene (minimum 98%) (Sigma-Aldrich, St. Louis, MO, USA), L-glutathione reduced (minimum 98%) (Sigma-Aldrich, Tokyo, Japan), 5,5′-Dithiobis (2-nitrobenzoic acid) (≥98%) (Sigma-Aldrich, St. Louis, MO, USA), acetylthiocholine iodide (≥98%) (Sigma-Aldrich, Gillingham, Kent, UK), and *p*-nitrophenyl phosphate (≥97%) (Sigma-Aldrich, Buchs, SG, Switzerland), were dissolved in a suitable solvent such as absolute ethanol, methanol and phosphate buffer. Fast Blue B Salt-Dye content (~95%) was purchased from Sigma-Aldrich, St. Louis, MO, USA. All other chemicals and reagents used were of analytical grade and procured from local agencies in Chiang Mai province, Thailand.

### 2.3. Mosquito Test Population and Colonization

The three mosquito populations used in this study were pyrethroid-susceptible and resistant strains of *Ae. aegypti*, comprising Muang Chiang Mai-susceptible (MCM-S), Pang Mai Dang-resistant (PMD-R), and Upakut-resistant (UPK-R), which were established from field larvae collected from clean stagnant water at various breeding sites in Chiang Mai province: Muang Chiang Mai district in 1995 [38], Ban Pang Mai Dang, Mae Tang district in 1997 [39], and Upakut Temple, Muang Chiang Mai district in 2006 [40], respectively. The MCM-S was resistant to DDT, but susceptible to pyrethroids such as permethrin and deltamethrin [31]. The PMD-R strain was resistant to both DDT and permethrin, but susceptible to deltamethrin [39,41,42,43], whereas the UPK-R strain was resistant to DDT, permethrin, and deltamethrin [40]. The PMD-R and UPK-R strains were reared under selection pressure by regular exposure to the WHO [44] discriminating dose (0.75% permethrin and 0.05% deltamethrin, respectively) in order to maintain the resistance level. All of the mosquitoes were maintained continually from their dates of collection in a climate-controlled insectary (25 ± 2 °C, 80 ± 10% Relative Humidity, and 14:10 h L/D photoperiod cycle) at the Department of Parasitology, Faculty of Medicine, CMU. The mosquito larvae were reared in plastic trays with tap water and fed on finely ground dog biscuits twice daily. The adults reared in humidified cages had continual access to 10% sucrose solution containing 10% multivitamin syrup, while females were blood-fed for egg production. Early 4th stage *Ae. aegypti* larvae of each strain were available for biochemical assays.

### 2.4. Determination of Lethal Threshold Time for the Lethal Effect of P. crispum Oil

Larvicidal bioassays, according to a slightly modified version of the WHO standard protocol [45], were performed to determine the threshold time for lethal effect of *P. crispum* oil on the three strains of *Ae. aegypti* under laboratory conditions. Four batches of 25 newly hatched 4th instar larvae were exposed to *P. crispum* essential oil solution at the median lethal concentrations (LC_50_) of 43.22, 44.50 and 44.03 ppm for MCM-S, PMD-R, and UPK-R strains, respectively, as estimated in an earlier study [31]. The control group comprised the larvae maintained in 0.4% DMSO-distilled water solution, whereas the untreated group was maintained in distilled water only. No food was provided for the larvae during the experiment. Bioassays were carried out under controlled conditions at 25 ± 2 °C and 80 ± 10% Relative Humidity, with four replicates. The lethal threshold time points for larvae were determined after recording the percentage of their mortality at time-intervals up until 24 h (0, 3, 6, 12, and 24 h). Exposure times that yielded larval mortality of 20–50% were considered as the threshold time for the lethal effect of *P. crispum* essential oil.

### 2.5. Preparation of Whole Body Homogenates Used for Enzyme Assay

After exposure to the test solutions at time-intervals up until 24 h (0, 3, 6 and 24 h), the larvae of treated, untreated and control groups obtained from each time-interval were washed with deionized water. These larvae were blotted with tissue paper for complete removal of adhered water and then kept in 1.5 mL tubes at −80 °C until their use for enzyme analysis. In order to prepare whole body homogenates, the stored larvae (2–10 individuals) of treated, untreated and control groups of each mosquito strain were homogenized separately in suitable buffer required for each enzyme assay. The whole body homogenates were centrifuged at 10,000× *g* at 4 °C for 20 min and the clear supernatants were used immediately for the determination of enzyme activity. All enzyme bioassays in this study were performed together with negative controls in the presence of homogenate buffer, but not enzyme, in the reactions.

### 2.6. Total Protein Assay

Total protein content in each larval homogenate was estimated according to the method of Bradford [46], with some minor modifications. Two hundred microliters of BIO Rad protein reagent diluted 1:4 in buffer (distilled water) were added to 10 µL of crude larval homogenate. The mixture was incubated in a 96-well microtiter plate at room temperature. Absorbance reading was taken at 595 nm after 5 min of reaction against homogenate buffer as a blank. Protein concentrations in mg/mL were calculated from a standard curve of bovine serum albumin (0–0.5 mg/mL). 

### 2.7. Glutathione S-Transferases (GSTs) Assay

GSTs activity was determined according to the modified method of Habig et al. [47]. Ten live larvae were homogenized in 200 µL of 0.1 M potassium phosphate buffer, pH 6.5. Two hundred microliters of GSH/CDNB working solution (10 mM reduced glutathione prepared in 0.1 M potassium phosphate buffer, pH 6.5 and 3 mM chlorodinitrobenzene diluted in methanol) were added to 10 µL of larval homogenate. Enzyme activity was measured at 340 nm, with the optimum time for reading of 2 min at room temperature. The GSTs activity was expressed as µmol CDNB conjugated/min/mg protein. 

### 2.8. α-/β-Esterases (α-/β-ESTs) Assays

The activity of *α*- and *β*-ESTs was determined according to the methodology of van Asperen [48], with slight modifications. Two live larvae were homogenized in 1 mL of 20 mM potassium phosphate buffer, pH 7.2. The larval homogenates were diluted with homogenization buffer at the ratio of 1:4 before measuring. Two hundred microliters of *α*- or *β*-naphthyl acetate solution (200 µL of 30 mM *α-* or *β*-NA in ethanol in 20 mL of 20 mM potassium phosphate buffer, pH 7.2) were added to 20 µL of larval homogenate. The mixture was incubated in a 96-well microtiter plate for 30 min at room temperature. The enzymatic reaction was stopped by the addition of 50 µL of Fast Blue B stain solution (22.5 mg of Fast Blue in 2.25 mL of distilled water, then 5.25 mL of 5% sodium lauryl sulphate diluted in distilled water). Replicate blanks contained 20 µL of homogenization buffer, 200 µL of substrate solution, and 50 µL of stop solution. Enzyme activity was read at 570 nm as an end point. The activities of *α*-/*β*-ESTs were calculated from the *α*- or *β*-naphthyl acetate standard curve. The activity of *α*-/*β*-ESTs was expressed as nmol of the *α*- or *β*-naphthol released/min/mg protein.

### 2.9. Acetylcholinesterase (AChE) Assay 

Evaluation of AChE activity was performed by following the modified method of Ellman et al. [49]. Five live larvae were homogenized in 250 µL of 0.1 M potassium phosphate buffer, pH 7.0 with 1 µM DL-dithiothreitol and 1% Triton X-100. An aliquot of 25 µL of larval homogenates was mixed successively with 155 µL of 0.65 mM dithiobis 2-nitrobenzoic acid solution and 25 µL of 10 mM acetylthiocholine iodide in a microliter plate. The absorbance reading was monitored at 405 nm after incubation for 5 min at room temperature. The AChE activity was reported as nmol of the acetylthiocholine hydrolyzed/min/mg protein.

### 2.10. Acid and Alkaline Phosphatases (ACP and ALP) Assays

The levels of ACP and ALP were measured by following the method of Asakura [50], with slight modifications. Ten live larvae were homogenized in 500 µL of appropriate homogenization buffer, which comprised 50 mM sodium acetate buffer, pH 4.0 and 50 mM Tris-HCl buffer, pH of 8.0, with 1 mM DL-dithiothreitol for the ACP and ALP assay, respectively. ACP activity was analyzed by mixing 10 µL of larval homogenate with 200 µL of substrate mixture containing 25 mM sodium acetate buffer, pH 4.0 and 6.25 mM *p*-nitrophenyl phosphate. For estimating ALP activity, buffer in substrate mixture also was substituted by 50 mM Tris-HCl buffer, pH 8.0. After incubation at 37 °C for 15 min in 96-well microtiter plates, the enzymatic reactions were terminated by adding 50 µL of 0.5 N NaOH. The absorbance was read at 405 nm and compared with the standard curve of known *p*-nitrophenol concentrations (0.0625–4 µg/µL). ACP/ALP activity was reported as nmol of the *p*-nitrophenol released/min/mg protein. 

### 2.11. Mixed-Function Oxidases (MFO) Assay

MFO activity was determined by using the heme-peroxidase assay of Brogdon et al. [51], with minor modifications. Ten live larvae were homogenized in 700 µL of 0.25 M sodium acetate buffer, pH 5.0. One hundred microliters of larval homogenate were mixed with 200 μL of substrate mixture and 6.3 mM TMBZ solution (0.01 g of 3,3′,5,5′-tetramethylbenzidine in 5 mL of absolute methanol mixed with 15 mL of 0.25 M sodium acetate buffer, pH 5.0, freshly prepared daily), in a 96-well microtiter plate. Finally, 25 μL of 3% H_2_O_2_ were added. The plate was incubated for 5 min at room temperature and then read at 630 nm by using a microplate reader (Spectra MR, DYNEX technologies, Chantilly, VA, USA). MFO values were compared with known concentrations of cytochrome c from horse heart type VI (0–1.4 ng/µL) and expressed as nmol of cytochrome c equivalent unit/mg protein.

### 2.12. Statistical Analysis

Data from biochemical assays were subjected to analysis of variance (One-way ANOVA) and expressed as a mean of 6–10 replicates using samples from different preparations. Significant differences between treatment groups among the three strains of mosquitoes were analyzed by Tukey’s HSD test (*p* < 0.05) using IBM SPSS statistics 24 (IBM Corp., Armonk, NY, USA). 

## 3. Results

### 3.1. Enzyme Activity Levels in the Three Strains of Ae. aegypti Prior to Treatment (0 h Time Point)

**Figure 1:**
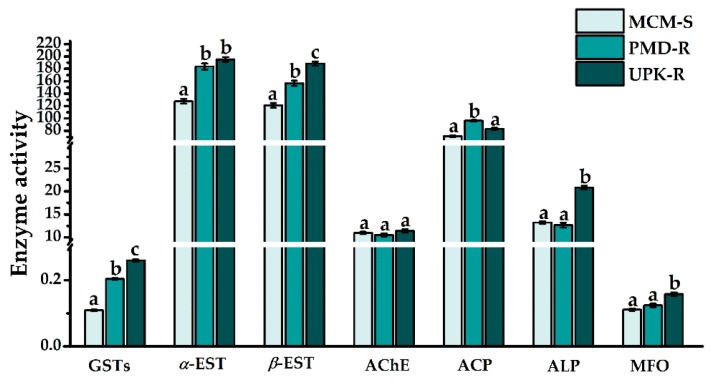
Enzyme activity in 4th instar larvae of the pyrethroid-susceptible (MCM-S) and resistant (PMD-R and UPK-R) *Ae. aegypti* prior to treatment (untreated group, 0 h time point). Each bar represents Mean ± SE of six or ten biological samples from different preparations. Bars with different letters on top indicate statistically significant differences among mosquito strains (Tukey’s HSD test, *p* < 0.05).

Prior to treatment with *P. crispum* oil, the baseline activity of some detoxifying enzymes, such as GSTs (F = 867.722, df = 2, *p* < 0.0001), *α*-EST (F = 74.345, df = 2, *p* < 0.0001), *β*-EST (F = 78.497, df = 2, *p* < 0.0001) and ACP (F = 82.421, df = 2, *p* < 0.0001) in resistant PMD-R and UPK-R larvae, was found to be significantly higher than that in susceptible MCM-S (Figure 1 and Table 1). A significant difference in highest GSTs and *β*-EST activity was recorded in UPK-R and PMD-R (*p* < 0.0001), with enzyme levels of 0.260 and 0.204 µmol, respectively, and 188.44 and 156.67 nmol, respectively. The maximum activity of *α*-EST was detected in UPK-R, followed by PMD-R and MCM-S at 195.23, 183.82 and 127.73 nmol, respectively. PMD-R showed the greatest activity of ACP, followed by UPK-R and MCM-S, with enzyme levels of 96.21, 83.29 and 71.31 nmol, respectively. UPK-R showed the significantly highest level of ALP activity (20.82 nmol), with the lowest one being detected in PMD-R (12.65 nmol), which was not statistically different (*p* = 0.571) from that detected in MCM-S (13.21 nmol). MFO activity also was higher in resistant UPK-R and PMD-R (0.158 and 0.124 nmol, respectively) than in MCM-S (0.111 nmol), but an insignificant difference of MFO level was observed between PMD-R and MCM-S. There was no significant difference among AChE activities estimated in UPK-R, PMD-R and MCM-S, with enzyme levels of 11.40, 10.51 and 10.96 nmol, respectively.

### 3.2. Threshold Time for the Lethal Effect of P. crispum Oil on Ae. aegypti Larvae

**Figure 2:**
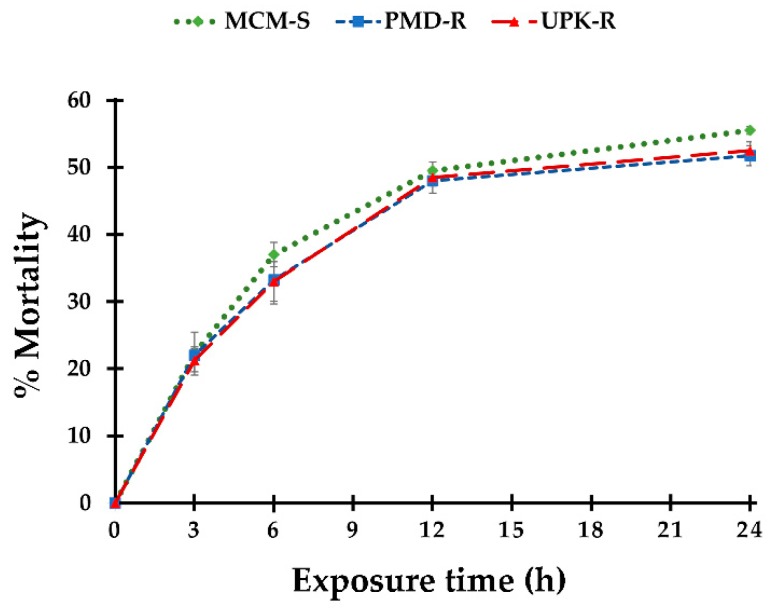
Threshold time for the lethal effect of exposure to LC_50_ of *P. crispum* oil on the three strains of *Ae. aegypti* larvae (43.22, 44.50 and 44.03 ppm for MCM-S, PMD-R and UPK-R strains, respectively). The error bars represent the standard errors of four replicates.

Determination of threshold time for the lethal effect of *P. crispum* oil on 4th instar *Ae. aegypti* larvae revealed that the mortality rate depended on the exposure period (Figure 2). Higher mortality was recorded with increasing exposure time. Approximately 20% of larvae were killed at 3 h and about 50% mortality was recorded at 24 h of treatment. All larvae maintained in control and untreated conditions survived and were still active during the experimental period. The results clearly indicated that exposure to LC_50_ of *P. crispum* oil for 3, 6, 12, and 24 h generated varied larval mortalities in the range of 20–50%, with no significant difference among three strains of *Ae. aegypti*. As larval mortalities from 12- and 24-h exposure were not significantly different, only three time points, such as 3, 6 and 24 h, were selected as suitable lethal threshold times for investigating the effect of *P. crispum* oil on biochemical constituents of *Ae. aegypti* larvae. 

### 3.3. Effects of P. crispum Oil on Biochemical Features of Ae. aegypti Larvae 

**Figure 3:**
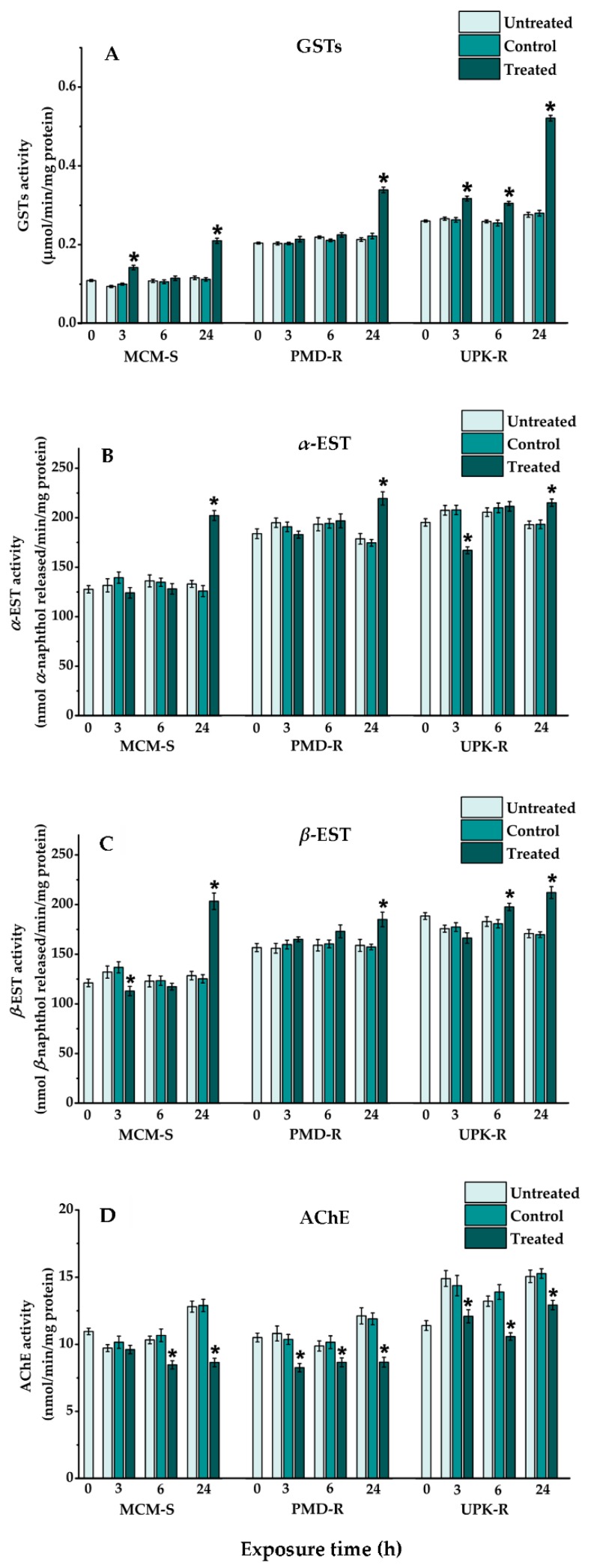
Activity levels of: GSTs (**A**); *α*-EST (**B**); *β*-EST (**C**); and AChE (**D**) in 4th instar larvae of the pyrethroid-susceptible (MCM-S) and resistant (PMD-R and UPK-R) *Ae. aegypti* after treatment with *P. crispum* oil (LC_50_) for 3, 6 and 24 h. Vertical bars represent standard error (±SE) of six or ten biological samples from different preparations. The asterisk shows statistically significant differences of means among the different groups (Tukey’s HSD test, *p* < 0.05).

**Figure 4:**
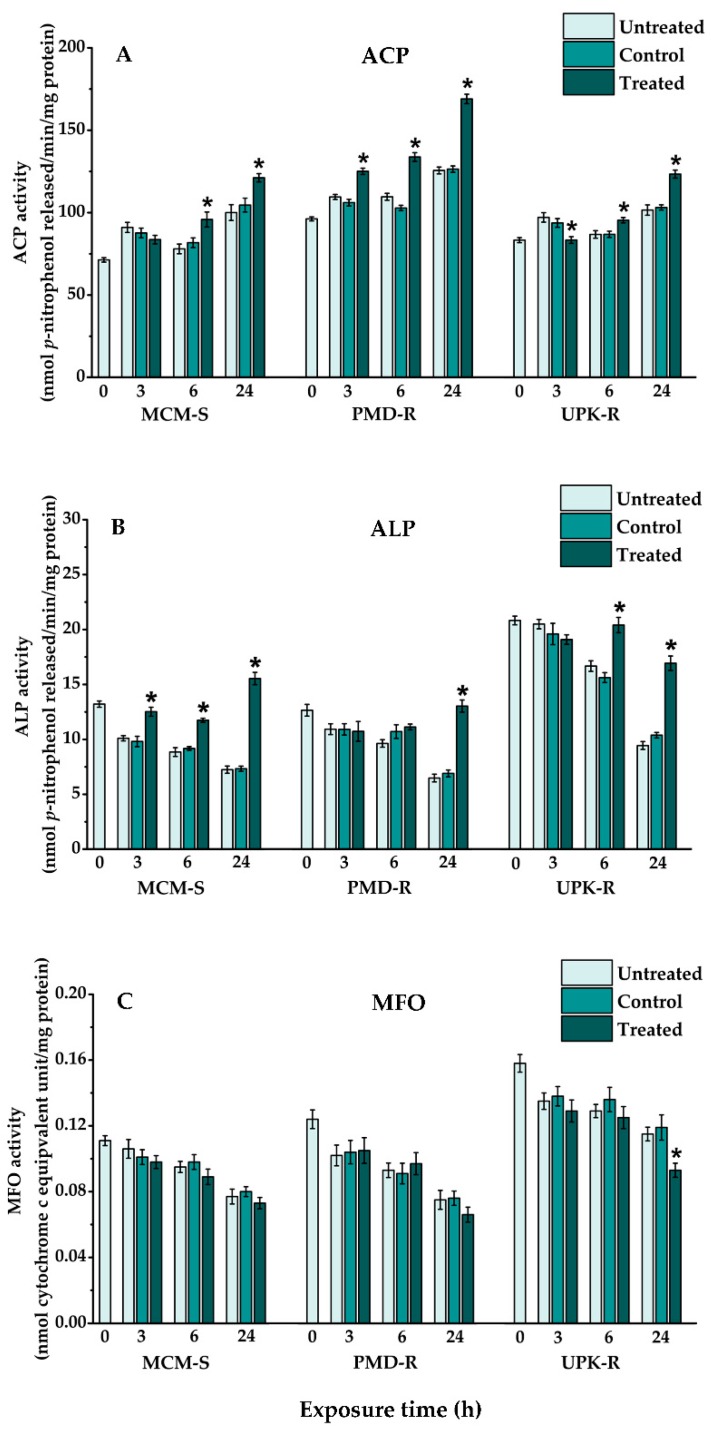
Activity levels of: ACP (**A**); ALP (**B**); and MFO (**C**) in 4th instar larvae of the pyrethroid-susceptible (MCM-S) and resistant (PMD-R and UPK-R) *Ae. aegypti* after treatment with *P. crispum* oil (LC_50_) for 3, 6 and 24 h. Vertical bars represent standard error (±SE) of six biological samples from different preparations. The asterisk shows statistically significant differences of means among the different groups (Tukey’s HSD test, *p* < 0.05).

The activity levels of all evaluated enzymes, including GSTs, *α*-/*β*-ESTs, AChE, ACP, ALP and MFO in the control and untreated groups of each strain of *Ae. aegypti*, were not significantly different (*p* > 0.05) at any of the time points (Figure 3 and Figure 4, Table 1). The enzyme profiles, except for *α*-/*β*-ESTs, also were altered similarly among the three strains of *Ae. aegypti* in these two groups during 24-h exposure. The activity level of GSTs in the control and untreated larvae of all strains was increased slightly from their baseline activity (0 h treatment). The increased AChE and ACP as well as decreased ALP and MFO activities in the control and untreated larvae of all strains were significantly different (*p* < 0.0001) from their baselines, except for the increased AChE activity in PMD-R.

After treatment with *P. crispum* oil for 3, 6 and 24 h, the activity levels of enzymes determined in the treated groups of each *Ae. aegypti* strain differed from those of the control and untreated groups (Figure 3 and Figure 4, Table 1). When comparing with the controls, the levels of GSTs in all strains of treated *Ae. aegypti* larvae were increased at every exposure time point (Figure 3A). Significant differences of GSTs activity between the treated and control groups were observed in MCM-S at 3 and 24 h, PMD-R at 24 h, and UPK-R at 3, 6 and 24 h of treatment (*p* < 0.0001). Although activity of *α*-/*β*-ESTs, ACP and ALP either increased or decreased in the three strains of treated larvae at all-time points during the exposure period, the levels of these enzymes, when detected at 24 h of treatment, eventually up-regulated when compared to those of the controls (Figure 3 and Figure 4, Table 1). Conversely, AChE activity in all strains of the treated larvae was reduced at every time point of exposure, when compared to that of the controls (Figure 3D). A statistically significant reduction of AChE activity was estimated in all strains of the treated larvae at every time point (MCM-S: 6 h, *p* = 0.000137; 24 h, *p* < 0.0001; PMD-R: 3 h, *p* = 0.002; 6 h, *p* = 0.025; 24 h, *p* = 0.000017; UPK-R: 3 h, *p* = 0.027; 6 h, *p* < 0.0001; 24 h, *p* = 0.002), apart from MCM-S at 3 h of treatment. Similar to AChE, the activity of MFO in all strains of treated larvae also decreased at every time point, except for those detected in PMD-R at 3- and 6-h exposure (Figure 4C).

## 4. Discussion

Plant essential oils have been recognized as potential sources of bioinsecticides used in agricultural pest and public health vector control programs. Among essential oils and ethanolic extracts of medicinal plants evaluated previously in the laboratory at Chiang Mai University, *P. crispum* essential oil exhibited larvicidal and adulticidal potential against both pyrethroid susceptible and resistant strains of *Ae. aegypti* [31]. With comparably mosquitocidal activity against pyrethroid susceptible and resistant *Ae. aegypti*, *P. crispum* essential oil became the candidate investigated herein for its effects on target mosquitoes, specifically in biochemical alterations. Three laboratory strains of *Ae. aegypti*, including MCM-S, PMD-R and UPK-R used in this study had been investigated by several investigators for status and mechanisms of insecticide resistance [39,40,42,43]. It was reported that while PMD-R showed resistance to permethrin, but susceptibility to deltamethrin, UPK-R proved to be resistant to both insecticides. However, our previous study revealed that both PMD-R and UPK-R were resistant to temephos, permethrin and deltamethrin in either the larval or adult stage, as compared to MCM-S [31]. Based on LC_50_ values obtained from a larvicidal bioassay, it is estimated that the susceptibility levels toward temephos, permethrin, and deltamethrin observed in PMD-R and UPK-R were lower than those of MCM-S by 1.9- and 1.9-fold; 4.1- and 286.0-fold; and 9.4- and 58.6-fold, respectively. According to Mazzarri and Georghiou [52], it can be clarified that PMD-R has low resistance to temephos and permethrin, but moderate resistance to deltamethrin, whereas UPK-R has low resistance to temephos, but high resistance to permethrin and deltamethrin. 

Biochemical profiles of *Ae. aegypti* larvae prior to treatment with *P. crispum* essential oil demonstrated higher levels of some detoxifying enzymes, such as GSTs, *α*-/*β*- ESTs, ACP and MFO in resistant PMD-R and/or UPK-R, than those in susceptible MCM-S. These findings were in agreement with those of many biochemical studies, which evaluated various pest species in either laboratory or field populations, and demonstrated increased levels of detoxifying enzymes such as GSTs [53,54], *α-*/*β*-ESTs [34,55,56,57,58], ACP [59,60], and MFO [61,62,63] in insecticide-resistant insects. Evidence of insecticide-resistant populations manifesting a higher level of defensive enzymes than susceptible ones, suggests that these enzymes possibly play a role in developing insect resistance. Many researchers have indicated that qualitative and/or quantitative changes of defensive enzymes may be an important process associated with the resistance mechanism [15,64], and elevation of enzyme activity implicate an enhancer in the tolerance of mosquitoes to insecticides [55,56,65,66]. In consequence, resistance to some insecticides has been linked to an increment in insecticide metabolism through the up-regulation or structural changes of detoxifying enzymes [67,68]. In this regard, resistance of PMD-R and UPK-R to temephos, permethrin, and deltamethrin may be due partly to alteration in the rate of insecticide detoxification via increased enzyme activity of the insect organism. Thus, the expression of detoxification enzymes such as GSTs, *α*-/*β*-ESTs, ACP and MFO might be used as an important biomarker of insecticide resistance in the insect population. 

Determining the threshold time for the lethal effect of *P. crispum* essential oil at LC_50_, demonstrated the exposure-time dependent efficacy against 4th instar of *Ae. aegypti*, with no significant difference of larval mortality among the three strains at any time point of treatment. Koodalingam et al. [37] investigated threshold time for the lethal effect of the soapnut, *Sapindus emarginatus*, on 4th instar larvae and pupae of *Ae. aegypti* by exposing them to lethal concentrations that produced 100% mortality within 24 h of exposure. Their study suggested that suitable lethal threshold times should be considered from the earliest time point of exposure, which generated the mortality outcome of 20–30% of test organisms upon their exposure to a specific test concentration. In this regard, 12 and 18 h of exposure were chosen as the threshold time points used for determining the lethal effect of soapnut extract on the larvae and pupae of *Ae. aegypti*, respectively. In order to gain more detailed insights of metabolic alterations affected by *P. crispum* oil in this study, sublethal concentrations (LC_50_) with different exposure periods, which generated a wide range of mortality, were applied on the three strains of *Ae. aegypti*. As a consequence, exposure times of 3, 6 and 24 h to LC_50_ of *P. crispum* oil producing 20–50% larval mortality were considered as appropriate threshold time points for determining the impact of this botanical oil on biochemical alterations in *Ae. aegypti* larvae. 

Evaluation of biochemical enzymes at three points of the lethal threshold times (3, 6 and 24 h of exposure) showed that after maintaining at a similar condition to the treated group, activities of all the evaluated enzymes, including GSTs, *α*-/*β*-ESTs, AChE, ACP, ALP and MFO observed in all strains of the control and untreated *Ae. aegypti* larvae, were not different at any of the time points. Furthermore, alterations of enzyme activity at 24-h intervals in these two groups were similar among the three strains of *Ae. aegypti*. Slight alterations either increased or decreased activity of GSTs and *α*-/*β*-ESTs in the control and untreated larvae of all mosquito strains, except for the decreased activity of *β*-EST in UPK-R, were different from their baseline activity (0 h treatment). However, apart from the AChE activity in PMD-R that insignificantly increased, significant changes in the enzyme activity of increased AChE and ACP as well as decreased ALP and MFO, were detected in the control and untreated larvae of all mosquito strains. It can be postulated that the activity of these defensive enzymes in the control and untreated groups was modulated during the normal developmental period (24 h of experiment), thus showing their importance in diverse physiological processes in the development of mosquito larvae, as reported in various insect [69,70].

Different levels of some detoxifying enzymes in this study also were detected between resistant PMD-R and UPK-R strains, in particular, higher levels of GSTs, *β*-EST, and MFO in UPK-R, either before or after treatment with *P. crispum* essential oil. As indicated previously, elevation of these enzymes presents insecticide resistance to pyrethroids [71,72,73]. Therefore, differences in enzyme activity between PMD-R and UPK-R probably contribute to their differential susceptibility or resistance to insecticides. In support of these findings, previous results [31] also proved that resistance was higher in UPK-R than in PMD-R by 69.8- and 6.2-fold for permethrin and deltamethrin, respectively. The activity levels of phosphatase enzymes that are responsible for detoxifying organophosphate insecticides, also were different between resistant PMD-R and UPK-R, which showed the highest level of ACP and ALP activity, respectively. Therefore, comparable resistance to organophosphate temephos (1.9-fold of MCM-S), as previously recorded in PMD-R and UPK-R [31], may be a result of balanced activity between two phosphatases, ACP, and ALP, in these resistant strains.

Upon exposing *Ae. aegypti* larvae to sublethal concentrations of *P. crispum* essential oil for 3, 6 and 24 h, enzymatic alterations of either up-regulated or down-regulated activity were detected in all of these mosquito strains. It was recognized that the activity levels of evaluated enzymes in the treated larvae were different from those of the control and untreated groups, depending on the type of enzyme, strain of mosquito, and time point after treatment. However, the enzyme expression patterns in the treated larvae altered similarly among the three strains of *Ae. aegypti* during 24 h of treatment, except for those of *β*-EST recorded at the 3- and 6-h time points as well as ACP and ALP at the 3-h time point. Although the baseline activity of most defensive enzymes was lower in susceptible MCM-S, than in resistant PMD-R and/or UPK-R, the higher up-regulation of *α*-/*β*-ESTs and ALP activity in MCM-S, after treatment with *P. crispum* essential oil, resulted in comparable enzyme levels in susceptible and resistant mosquitoes at the 24-h time point. Unlike, the similar elevation rates of GSTs and ACP activity in susceptible and resistant strains of mosquitoes contributed to lower levels of these enzymes in treated MCM-S, as compared to those of PMD-R and/or UPK-R at the 24-h time point. However, the activities of GSTs, *α*-/*β*-ESTs, ACP, and ALP in treated larvae of all the *Ae. aegypti* strains were found to elevate after 24 h of treatment. These findings corresponded to those of many studies that demonstrated increased levels of GSTs [27,74,75], *α*-/*β*-ESTs [66,76,77,78], and ACP and ALP [69,79,80] in plant product-treated insects. The prominent alterations of these enzymes possibly correlate to the phytochemicals present in plant products, and their increased expression might suggest a probable role in detoxifying tested biocides into nontoxic substances [81,82].

GSTs, esterases (*α*-/*β*-ESTs), and phosphatases (ACP and ALP) are enzymes involved in the transformation and/or elimination of endogenous and exogenous compounds through several metabolic pathways in insects [34,35,36,37]. In addition to catalyzing the conjugation of glutathione with electrophilic compounds, GSTs also act as ligand-binding proteins that lead to the sequestration of xenobiotics [83,84,85,86]. Esterases are classified as hydrolases, a large and diverse group of enzymes that catalyze ester bonds found in a wide range of insecticides such as organophosphates, carbamates, and pyrethroids [87]. Phosphatases play an important role in diverse physiological processes and act as hydrolytic enzymes that catalyze removal of phosphate groups by hydrolyzing phosphate ester bonds, which are found in organophosphate insecticides [88,89]. Regarding the results obtained from the present and previous study, the increased activity of defensive enzymes, including GSTs, *α*-/*β*-ESTs, ACP, and ALP, in *P. crispum* oil-treated larvae of all the *Ae. aegypti* strains, indicated their possible role in metabolizing oil components. Elevation of these enzymes, observed during treatment with *P. crispum* essential oil, may be attributed to their overproduction. It is hypothesized that some oil constituents that enter the body of insects could be metabolized directly by these detoxifying enzymes, while the rest of the tested oil that binds to receptors could induce more enzyme production [66]. 

AChE and MFO activity in all of the treated *Ae. aegypti* strains in this study reduced when compared to the controls, as recorded after exposure to *P. crispum* essential oil for 24 h. Similar results of reduced activity of AChE [37,90,91] and MFO [22,26,92,93,94] were observed in several insect species exposed to different botanical products. These findings were contrary to those in many studies, which demonstrated biochemical responses with elevation of AChE or MFO activity after treatment with either synthetic substances or plant products. High activity of AChE and MFO was observed in all developmental stages of *Culex pipiens* treated with agricultural waste extracts, black and white liquors [95]. Evaluation of resistance to treatment with temephos and Pb-CVO (plant oil product) was carried out in a field collected wild strain (WS) and susceptible laboratory strain (LS) of *Ae. aegypti* [96]. The results demonstrated increased levels of cytochrome P450 monooxygenases (CYP450) in both strains of Pb-CVO-treated *Ae. aegypti*. However, CYP450 levels were increased in the LS, but decreased in the WS after treatment with temephos. Up-regulation of CYP450 level also was observed in *Ae. aegypti* larvae treated with the lethal concentration of *Alangium salvifolium* methanolic leaf extract [27]. The effects of girgensohnine alkaloid analog (DPPA) and *Cymbopogon flexuosus* essential oil (CFEO) on the behavior of detoxifying enzymes were evaluated in Rockefeller laboratory (Rock) and Santander wild (WSant) strains of *Ae. aegypti* [97]. It was found that the AChE activity in Rock and WSant was not affected by the evaluated dosages of DPPA and CFEO (*p* > 0.05), while MFO activity was significantly influenced by all of the CFEO dosages in WSant (*p* < 0.05). It was noticeable from these findings that differences in biochemical response in the target insects may be influenced by a variety of factors such as type of enzyme, type, and dosage of test substance, as well as developmental stages, species, and susceptibility of the insect. 

AChE, a serine esterase (hydrolase) mainly found in nerve synapses, is a key enzyme that terminates nerve impulses by catalyzing hydrolysis of the neurotransmitter acetylcholine in the nervous system [98]. AChE also is the primary target of organophosphorus and carbamate compounds, which inhibit this enzyme, resulting in a general nervous system failure [99,100,101]. MFO is a family of enzymes induced when organisms are exposed to a variety of xenobiotics, and also is the most important enzyme involved in detoxifying pyrethroid and organophosphorus insecticides [102,103,104]. The increase and decrease of AChE and MFO after exposure to natural or synthetic compounds would probably result from the impact of these substances on insect metabolism by enzyme regulations, either inhibiting or stimulating enzyme activity. This appears to show biochemical interruption of diversified physiological mechanisms of insects [69]. Decreased AChE and MFO activity in all of the *P. crispum* oil-treated larvae strains in this study led to the postulation that these enzymes play no or a minimal role in metabolizing the tested oil, but they are a possible target of oil components. Due to the important role in various physiological processes, reduction of AChE and MFO, resulting from inhibitory effect of *P. crispum* essential oil, probably generates physiological disturbance and eventual death of *Ae. aegypti* larvae. 

The comparatively pronounced larvicidal capability in pyrethroid susceptible MCM-S and resistant PMD-R and UPK-R of *Ae. aegypti* [31], and the inhibitory effect on AChE and MFO, emphasizes *P. crispum* essential oil as a qualified alternative for further development and application in current and future mosquito control programs. While AChE inhibitor affects nerve transmission, inhibition of MFO results in insecticide detoxification. Of interest, reduction of MFO activity in resistant UPK-R (21.8%) and PMD-R (13.2%), after treatment with *P. crispum* essential oil, sparked the idea of using this plant oil in managing mosquito resistance by combining it with existing conventional insecticides that are degraded by MFO such as pyrethroids and organophosphates, and this topic needs to be studied further. The combination of *P. crispum* essential oil and chemical compounds is a targetable prospect for synergist addition, which not only decreases dose application of insecticides, but also increases their mosquitocidal efficacy. As the target of MFO, pyrethroids and organophosphates can be detoxified by this enzyme, the lowered level of MFO leads to lowered insecticide detoxification, resulting in enhanced insecticidal efficacy. The synergism potential from MFO inhibition of *P. crispum* essential oil, which increased the toxic effects of pyrethroids and organophosphates, might be helpful in fighting against resistant mosquitoes, which have a high level of MFO activity. Furthermore, as *P. crispum* essential oil is a natural product comprising varied and complex active principles, with multiple modes of action through different targets such as AChE and MFO, a combination of *P. crispum* oil-insecticide may contribute to prolong the generation of resistance in vector populations. In consequence, this cumulative strategy may improve the performance of integrated mosquito management programs.

## 5. Conclusions

Biochemical profiles in the pyrethroid susceptible and resistant strains of *Ae. aegypti* larvae before and after exposure to *P. crispum* oil showed alterations of key enzymes responsible for xenobiotic detoxification, including GSTs, *α*-/*β*-ESTs, AChE, ACP, ALP, and MFO. The recognizable larvicidal capability on pyrethroid resistant *Ae*. *aegypti* and the inhibitory effect on AChE and MFO emphasize the potential of *P. crispum* oil as an attractive alternative for the management of mosquito resistance in current and future control programs.

## Figures and Tables

**Table 1:** Enzyme activity in pyrethroid-susceptible (MCM-S) and resistant (PMD-R and UPK-R) strains of *Ae. aegypti* after treatment with LC_50_ of *P. crispum* oil.

Enzyme		Enzyme Activity (Mean ± SE) *		
		MCM-S	PMD-R	UPK-R
GSTs ^1^				
0 h	Untreated	0.109 ± 0.002 ^a,A^	0.204 ± 0.002 ^a,B^	0.260 ± 0.003 ^a,C^
24 h	Untreated	0.116 ± 0.004 ^a,A^	0.227 ± 0.004 ^a,B^	0.276 ± 0.006 ^a,b,C^
	Control	0.112 ± 0.004 ^a,A^	0.242 ± 0.006 ^a,B^	0.280 ± 0.007 ^b,C^
	Treated	0.210 ± 0.007 ^b,A^	0.442 ± 0.021 ^b,B^	0.521 ± 0.007 ^c,C^
*α*-EST ^2^				
0 h	Untreated	127.73 ± 3.73 ^a,A^	183.82 ± 4.94 ^a,B^	195.23 ± 3.79 ^a,B^
24 h	Untreated	133.10 ± 3.52 ^a,A^	178.64 ± 5.35 ^a,B^	192.88 ± 3.79 ^a,B^
	Control	125.92 ± 5.56 ^a,A^	174.59 ± 3.40 ^a,B^	193.33 ± 4.40 ^a,C^
	Treated	202.19 ± 5.08 ^b,A^	219.44 ± 6.71 ^b,A^	215.14 ± 3.67 ^b,A^
*β*-EST ^3^				
0 h	Untreated	121.07 ± 3.84 ^a,A^	156.67 ± 4.10 ^a,B^	188.44 ± 3.44 ^a,C^
24 h	Untreated	128.42 ± 4.33 ^a,A^	158.87 ± 6.04 ^a,B^	170.79 ± 4.15 ^b,B^
	Control	125.28 ± 4.16 ^a,A^	157.25 ± 2.83 ^a,B^	169.66 ± 2.92 ^b,C^
	Treated	203.34 ± 8.31 ^b,A,B^	185.03 ± 7.41 ^b,A^	212.09 ± 5.99 ^c,B^
AChE ^4^				
0 h	Untreated	10.96 ± 0.24 ^a,A^	10.51 ± 0.33 ^a,A^	11.40 ± 0.36 ^a,A^
24 h	Untreated	12.81 ± 0.42 ^b,A^	12.12 ± 0.60 ^a,A^	15.06 ± 0.46 ^b,B^
	Control	12.90 ± 0.46 ^b,A^	11.90 ± 0.44 ^a,A^	15.27 ± 0.35 ^b,B^
	Treated	8.65 ± 0.32 ^c,A^	8.67 ± 0.37 ^b,A^	12.93 ± 0.35 ^c,B^
ACP ^5^				
0 h	Untreated	71.31 ± 1.34 ^a,A^	96.21 ± 1.21 ^a,C^	83.29 ± 1.55 ^a,B^
24 h	Untreated	100.02 ± 4.81 ^b,A^	125.55 ± 2.07 ^b,B^	101.57 ± 3.14 ^b,A^
	Control	104.56 ± 4.20 ^b,A^	126.39 ± 1.94 ^b,B^	103.13 ± 1.60 ^b,A^
	Treated	121.12 ± 2.52 ^c,A^	168.97 ± 2.85 ^c,B^	123.35 ± 2.41^c,A^
ALP ^6^				
0 h	Untreated	13.21 ± 0.28 ^a,A^	12.65 ± 0.54 ^a,A^	20.82 ± 0.40 ^a,B^
24 h	Untreated	7.24 ± 0.33 ^b,A^	6.48 ± 0.35 ^b,A^	9.44 ± 0.36 ^b,B^
	Control	7.33 ± 0.23 ^b,A^	6.90 ± 0.31 ^b,A^	10.38 ± 0.25 ^b,B^
	Treated	15.54 ± 0.56 ^c,A^	13.03 ± 0.55 ^a,B^	16.93 ± 0.65 ^c,A^
MFO ^7^				
0 h	Untreated	0.111 ± 0.003 ^a,A^	0.124 ± 0.006 ^a,A^	0.158 ± 0.005 ^a,B^
24 h	Untreated	0.077 ± 0.005 ^b,A^	0.075 ± 0.006 ^b,A^	0.115 ± 0.004 ^b,c,B^
	Control	0.080 ± 0.003 ^b,A^	0.076 ± 0.004 ^b,A^	0.119 ± 0.008 ^b,B^
	Treated	0.073 ± 0.003 ^b,A^	0.066 ± 0.005 ^b,A^	0.093 ± 0.004 ^c,B^

* Values followed by different lowercase letters in a column and uppercase letters in a row are significantly different (Tukey’s HSD test, *p* < 0.05); ^1^ GSTs activity was expressed as µmol/min/mg protein; ^2^ *α*-EST activity was expressed as nmol/*α*-naphthol released/min/mg protein; ^3^ *β*-EST activity was expressed as nmol/*β*-naphthol released/min/mg protein; ^4^ AChE activity was expressed as nmol/min/mg protein; ^5^ ACP activity was expressed as nmol *p*-nitrophenol released/min/mg protein; ^6^ ALP activity was expressed as nmol *p*-nitrophenol released/min/mg protein; ^7^ MFO activity was expressed as nmol cytochrome c equivalent unit/mg protein.
